# Tetra-μ-acetato-κ^8^ *O*:*O*′-bis­{[4-methyl-2-(*m*-tolyl­amino)pyridine-κ*N*]copper(II)}

**DOI:** 10.1107/S1600536809055858

**Published:** 2010-01-16

**Authors:** Zainal Abidin Fairuz, Zaharah Aiyub, Zanariah Abdullah, Seik Weng Ng

**Affiliations:** a Department of Chemistry, University of Malaya, 50603 Kuala Lumpur, Malaysia

## Abstract

In the crystal structure of the title binuclear complex, [Cu_2_(CH_3_COO)_4_(C_13_H_14_N_2_)_2_], the four acetate groups each bridge a pair of Cu^II^ atoms. The coordination of the metal atoms is distorted square-pyramidal, with the bonding O atoms comprising a square basal plane and the coordinating N atom of the *N*-heterocycle occupying the apical position. In the two *N*-hetercycle ligands, the benzene rings are twisted with respect to the pyridine rings, making dihedral angles of 53.1 (2) and 54.2 (2)°. Intra­molecular N—H⋯O hydrogen bonding is present between the imino and carb­oxy groups. The crystal studied was a non-merohedral twin with a minor twin component of 21.4%.

## Related literature

For the 2-(*m*-tolyl­amino)pyridine adduct, see: Fairuz *et al.* (2009 ▶). For the treatment of diffraction data of twinned crystals, see: Spek (2003 ▶).
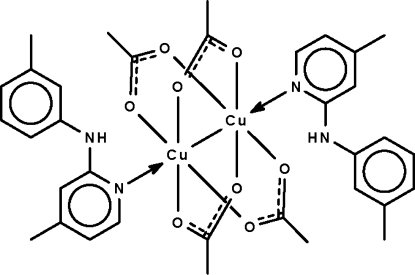


## Experimental

### 

#### Crystal data

[Cu_2_(C_2_H_3_O_2_)_4_(C_13_H_14_N_2_)_2_]
*M* *_r_* = 759.78
Triclinic, 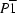

*a* = 8.2489 (4) Å
*b* = 14.2110 (7) Å
*c* = 16.4484 (8) Å
α = 107.798 (1)°
β = 101.971 (1)°
γ = 97.661 (1)°
*V* = 1755.45 (15) Å^3^
*Z* = 2
Mo *K*α radiation
μ = 1.27 mm^−1^
*T* = 295 K
0.40 × 0.10 × 0.10 mm

#### Data collection

Bruker SMART APEX diffractometer
Absorption correction: multi-scan (*SADABS*; Sheldrick, 1996 ▶) *T*_min_ = 0.631, *T*_max_ = 0.884
21605 measured reflections
7963 independent reflections
6719 reflections with *I* > 2σ(*I*)
*R*_int_ = 0.030

#### Refinement

*R*[*F* ^2^ > 2σ(*F* ^2^)] = 0.066
*wR*(*F* ^2^) = 0.231
*S* = 1.12
7963 reflections
442 parameters
H-atom parameters constrained
Δρ_max_ = 1.01 e Å^−3^
Δρ_min_ = −0.91 e Å^−3^

### 

Data collection: *APEX2* (Bruker, 2008 ▶); cell refinement: *SAINT* (Bruker, 2008 ▶); data reduction: *SAINT*; program(s) used to solve structure: *SHELXS97* (Sheldrick, 2008 ▶); program(s) used to refine structure: *SHELXL97* (Sheldrick, 2008 ▶); molecular graphics: *X-SEED* (Barbour, 2001 ▶); software used to prepare material for publication: *publCIF* (Westrip, 2010 ▶).

## Supplementary Material

Crystal structure: contains datablocks global, I. DOI: 10.1107/S1600536809055858/xu2709sup1.cif

Structure factors: contains datablocks I. DOI: 10.1107/S1600536809055858/xu2709Isup2.hkl

Additional supplementary materials:  crystallographic information; 3D view; checkCIF report

## Figures and Tables

**Table 1:** Selected bond lengths (Å)

Cu1—O1	1.985 (4)
Cu1—O4	1.961 (5)
Cu1—O6	1.963 (4)
Cu1—O8	1.956 (5)
Cu1—N1	2.215 (5)
Cu1—Cu	2.6576 (9)
Cu2—O2	1.968 (4)
Cu2—O3	2.001 (5)
Cu2—O5	1.957 (5)
Cu2—O7	1.981 (5)
Cu2—N3	2.208 (5)

**Table 2:** Hydrogen-bond geometry (Å, °)

*D*—H⋯*A*	*D*—H	H⋯*A*	*D*⋯*A*	*D*—H⋯*A*
N2—H2⋯O1	0.86	1.99	2.830 (6)	165
N4—H4⋯O3	0.86	2.18	2.964 (8)	152

**Fig. 1.**
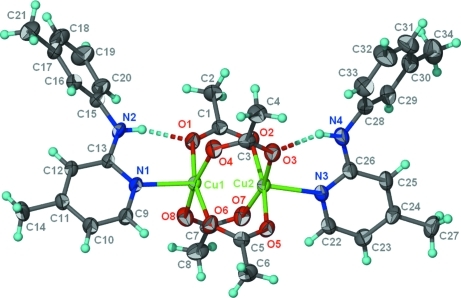
Thermal ellipsoid plot (Barbour, 2001) of Cu2(C2H3O2)2(C13H14N2)2 at the 50% probability level; hydrogen atoms are drawn as spheres of arbitrary radius.

## supplementary crystallographic information

### Experimental

Copper acetate (0.1 g, 0.5 mmol) was dissolved in acetonitrile (5 ml). The solution was mixed with a solution of 4-methyl-2-(*m*-tolylamino)pyridine (0.2 g, 1.1 mmol) dissolved in acetonitrile (15 ml). The green precipitate that formed was recrystallized from acetonitrile to give greenish-blue crystals.

### Refinement

Carbon-bound H-atoms were placed in calculated positions (C–H 0.93–0.96 Å) and were included in the refinement in the riding model approximation, with *U*(H) set to 1.2–1.5*U*(C). The imino H-atoms were similarly treated. The final difference Fourier map had a peak in the vicinity of H14*b*.

The structure is a non-merohedral twin. Another crystal that was examined also show twinning. The diffraction data were detwinned by using *PLATON* (Spek, 2003).

### Crystal data

**Table:**

[Cu_2_(C_2_H_3_O_2_)_4_(C_13_H_14_N_2_)_2_]	*Z* = 2
*M**_r_* = 759.78	*F*(000) = 788
Triclinic, *P*1	*D*_x_ = 1.437 Mg m^−^^3^
Hall symbol: -P 1	Mo *K*α radiation, λ = 0.71073 Å
*a* = 8.2489 (4) Å	Cell parameters from 9994 reflections
*b* = 14.2110 (7) Å	θ = 2.4–28.3°
*c* = 16.4484 (8) Å	µ = 1.27 mm^−^^1^
α = 107.798 (1)°	*T* = 295 K
β = 101.971 (1)°	Prism, blue
γ = 97.661 (1)°	0.40 × 0.10 × 0.10 mm
*V* = 1755.45 (15) Å^3^	

### Data collection

**Table:**

Bruker SMART APEX diffractometer	7963 independent reflections
Radiation source: fine-focus sealed tube	6719 reflections with *I* > 2σ(*I*)
graphite	*R*_int_ = 0.030
ω scans	θ_max_ = 27.5°, θ_min_ = 1.4°
Absorption correction: multi-scan (*SADABS*; Sheldrick, 1996)	*h* = −10→10
*T*_min_ = 0.631, *T*_max_ = 0.884	*k* = −18→18
21605 measured reflections	*l* = −21→21

### Refinement

**Table:**

Refinement on *F*^2^	Primary atom site location: structure-invariant direct methods
Least-squares matrix: full	Secondary atom site location: difference Fourier map
*R*[*F*^2^ > 2σ(*F*^2^)] = 0.066	Hydrogen site location: inferred from neighbouring sites
*wR*(*F*^2^) = 0.231	H-atom parameters constrained
*S* = 1.12	*w* = 1/[σ^2^(*F*_o_^2^) + (0.0917*P*)^2^ + 6.9746*P*] where *P* = (*F*_o_^2^ + 2*F*_c_^2^)/3
7963 reflections	(Δ/σ)_max_ = 0.001
442 parameters	Δρ_max_ = 1.01 e Å^−^^3^
0 restraints	Δρ_min_ = −0.91 e Å^−^^3^

### Fractional atomic coordinates and isotropic or equivalent isotropic displacement parameters (Å^2^)

**Table:**

	*x*	*y*	*z*	*U*_iso_*/*U*_eq_	
Cu1	0.86052 (8)	0.69737 (5)	0.46112 (4)	0.03279 (19)	
Cu2	0.79744 (9)	0.63662 (5)	0.28585 (4)	0.0358 (2)	
N1	0.9016 (6)	0.7346 (4)	0.6058 (3)	0.0375 (10)	
N2	0.6928 (7)	0.8250 (4)	0.6207 (3)	0.0477 (13)	
H2	0.6773	0.8115	0.5647	0.057*	
N3	0.7634 (7)	0.5752 (4)	0.1414 (3)	0.0446 (12)	
N4	0.7788 (10)	0.7301 (4)	0.1219 (4)	0.0630 (18)	
H4	0.7981	0.7548	0.1785	0.076*	
O1	0.6452 (6)	0.7438 (4)	0.4354 (3)	0.0503 (11)	
O2	0.6044 (6)	0.7040 (4)	0.2906 (3)	0.0508 (11)	
O3	0.9404 (6)	0.7729 (4)	0.3116 (3)	0.0502 (11)	
O4	0.9801 (6)	0.8244 (3)	0.4577 (3)	0.0503 (11)	
O5	1.0020 (6)	0.5821 (4)	0.3059 (3)	0.0494 (11)	
O6	1.0653 (6)	0.6426 (4)	0.4532 (3)	0.0515 (12)	
O7	0.6626 (6)	0.5144 (4)	0.2927 (3)	0.0528 (12)	
O8	0.7303 (6)	0.5625 (4)	0.4395 (3)	0.0515 (11)	
C1	0.5674 (8)	0.7429 (5)	0.3606 (4)	0.0399 (12)	
C2	0.4178 (10)	0.7926 (6)	0.3580 (5)	0.0582 (19)	
H2A	0.3212	0.7470	0.3134	0.087*	
H2B	0.4435	0.8530	0.3442	0.087*	
H2C	0.3930	0.8096	0.4146	0.087*	
C3	0.9957 (7)	0.8375 (4)	0.3880 (4)	0.0403 (13)	
C4	1.0904 (10)	0.9404 (5)	0.3968 (6)	0.0580 (18)	
H4A	1.1758	0.9680	0.4522	0.087*	
H4B	1.0124	0.9847	0.3948	0.087*	
H4C	1.1430	0.9339	0.3491	0.087*	
C5	1.0947 (7)	0.5954 (5)	0.3810 (4)	0.0400 (12)	
C6	1.2513 (9)	0.5518 (6)	0.3873 (5)	0.0572 (18)	
H6A	1.3469	0.6033	0.4267	0.086*	
H6B	1.2708	0.5273	0.3297	0.086*	
H6C	1.2362	0.4971	0.4096	0.086*	
C7	0.6589 (8)	0.5004 (5)	0.3637 (5)	0.0437 (14)	
C8	0.5583 (10)	0.4005 (5)	0.3577 (6)	0.0594 (19)	
H8A	0.5146	0.4097	0.4087	0.089*	
H8B	0.6304	0.3526	0.3554	0.089*	
H8C	0.4658	0.3758	0.3051	0.089*	
C9	1.0151 (8)	0.6885 (5)	0.6409 (4)	0.0458 (14)	
H9	1.0743	0.6525	0.6042	0.055*	
C10	1.0498 (8)	0.6906 (5)	0.7262 (4)	0.0474 (15)	
H10	1.1312	0.6579	0.7466	0.057*	
C11	0.9612 (8)	0.7425 (5)	0.7821 (4)	0.0416 (13)	
C12	0.8430 (8)	0.7908 (5)	0.7484 (4)	0.0402 (13)	
H12	0.7829	0.8269	0.7845	0.048*	
C13	0.8138 (7)	0.7852 (4)	0.6601 (4)	0.0331 (11)	
C14	0.9903 (11)	0.7440 (7)	0.8763 (5)	0.063 (2)	
H14A	0.8965	0.7633	0.8987	0.095*	
H14B	1.0929	0.7918	0.9123	0.095*	
H14C	0.9999	0.6779	0.8777	0.095*	
C15	0.5887 (8)	0.8861 (5)	0.6609 (4)	0.0404 (13)	
C16	0.6562 (9)	0.9697 (5)	0.7366 (4)	0.0438 (13)	
H16	0.7715	0.9842	0.7644	0.053*	
C17	0.5539 (10)	1.0326 (5)	0.7719 (4)	0.0511 (16)	
C18	0.3834 (11)	1.0100 (7)	0.7289 (6)	0.066 (2)	
H18	0.3136	1.0516	0.7515	0.079*	
C19	0.3146 (11)	0.9265 (7)	0.6527 (7)	0.072 (2)	
H19	0.1999	0.9125	0.6239	0.086*	
C20	0.4190 (9)	0.8643 (6)	0.6201 (5)	0.0518 (16)	
H20	0.3733	0.8070	0.5699	0.062*	
C21	0.6282 (15)	1.1255 (7)	0.8540 (6)	0.080 (3)	
H21A	0.5907	1.1829	0.8436	0.120*	
H21B	0.7497	1.1377	0.8678	0.120*	
H21C	0.5912	1.1148	0.9027	0.120*	
C22	0.7531 (11)	0.4764 (5)	0.1082 (4)	0.0535 (17)	
H22	0.7495	0.4395	0.1460	0.064*	
C23	0.7473 (11)	0.4248 (5)	0.0217 (5)	0.0571 (18)	
H23	0.7372	0.3551	0.0019	0.068*	
C24	0.7568 (9)	0.4778 (5)	−0.0355 (4)	0.0495 (15)	
C25	0.7643 (11)	0.5799 (6)	−0.0030 (5)	0.0568 (18)	
H25	0.7676	0.6179	−0.0399	0.068*	
C26	0.7669 (9)	0.6269 (5)	0.0855 (4)	0.0461 (14)	
C27	0.7588 (13)	0.4265 (6)	−0.1297 (5)	0.069 (2)	
H27A	0.7902	0.3625	−0.1358	0.104*	
H27B	0.8394	0.4683	−0.1454	0.104*	
H27C	0.6480	0.4159	−0.1681	0.104*	
C28	0.7628 (9)	0.7992 (5)	0.0763 (4)	0.0479 (15)	
C29	0.6370 (9)	0.7780 (6)	0.0001 (5)	0.0509 (16)	
H29	0.5640	0.7149	−0.0241	0.061*	
C30	0.6161 (10)	0.8499 (6)	−0.0424 (5)	0.0590 (18)	
C31	0.7265 (13)	0.9410 (7)	−0.0052 (7)	0.075 (2)	
H31	0.7166	0.9898	−0.0318	0.090*	
C32	0.8540 (15)	0.9624 (7)	0.0719 (7)	0.085 (3)	
H32	0.9281	1.0252	0.0962	0.102*	
C33	0.8715 (10)	0.8916 (6)	0.1122 (5)	0.0600 (19)	
H33	0.9569	0.9064	0.1638	0.072*	
C34	0.4771 (12)	0.8248 (9)	−0.1222 (6)	0.083 (3)	
H34A	0.5157	0.8512	−0.1636	0.124*	
H34B	0.3845	0.8540	−0.1068	0.124*	
H34C	0.4400	0.7527	−0.1485	0.124*	

### Atomic displacement parameters (Å^2^)

**Table:**

	*U*^11^	*U*^22^	*U*^33^	*U*^12^	*U*^13^	*U*^23^
Cu1	0.0316 (3)	0.0375 (4)	0.0286 (3)	0.0092 (3)	0.0081 (3)	0.0095 (3)
Cu2	0.0371 (4)	0.0404 (4)	0.0286 (3)	0.0096 (3)	0.0090 (3)	0.0092 (3)
N1	0.040 (3)	0.043 (3)	0.031 (2)	0.015 (2)	0.0123 (19)	0.009 (2)
N2	0.060 (3)	0.060 (3)	0.028 (2)	0.035 (3)	0.016 (2)	0.010 (2)
N3	0.057 (3)	0.046 (3)	0.031 (2)	0.014 (2)	0.008 (2)	0.013 (2)
N4	0.111 (6)	0.044 (3)	0.033 (3)	0.016 (3)	0.017 (3)	0.013 (2)
O1	0.050 (3)	0.071 (3)	0.035 (2)	0.029 (2)	0.0111 (19)	0.018 (2)
O2	0.042 (2)	0.073 (3)	0.039 (2)	0.025 (2)	0.0098 (19)	0.017 (2)
O3	0.057 (3)	0.046 (2)	0.045 (3)	0.003 (2)	0.016 (2)	0.014 (2)
O4	0.060 (3)	0.042 (2)	0.043 (2)	0.000 (2)	0.012 (2)	0.0105 (19)
O5	0.050 (3)	0.062 (3)	0.043 (2)	0.026 (2)	0.019 (2)	0.017 (2)
O6	0.044 (2)	0.075 (3)	0.040 (2)	0.029 (2)	0.0148 (19)	0.016 (2)
O7	0.057 (3)	0.046 (3)	0.046 (3)	−0.001 (2)	0.016 (2)	0.007 (2)
O8	0.058 (3)	0.047 (3)	0.044 (2)	0.000 (2)	0.008 (2)	0.017 (2)
C1	0.037 (3)	0.044 (3)	0.043 (3)	0.014 (2)	0.012 (2)	0.019 (3)
C2	0.055 (4)	0.076 (5)	0.061 (4)	0.038 (4)	0.021 (3)	0.034 (4)
C3	0.034 (3)	0.037 (3)	0.052 (4)	0.008 (2)	0.015 (3)	0.016 (3)
C4	0.059 (4)	0.046 (4)	0.073 (5)	0.004 (3)	0.028 (4)	0.023 (3)
C5	0.035 (3)	0.040 (3)	0.047 (3)	0.008 (2)	0.013 (3)	0.017 (3)
C6	0.045 (4)	0.069 (5)	0.066 (5)	0.025 (3)	0.021 (3)	0.026 (4)
C7	0.032 (3)	0.038 (3)	0.059 (4)	0.007 (2)	0.014 (3)	0.013 (3)
C8	0.054 (4)	0.039 (3)	0.082 (5)	0.005 (3)	0.022 (4)	0.015 (3)
C9	0.045 (3)	0.062 (4)	0.036 (3)	0.024 (3)	0.014 (3)	0.017 (3)
C10	0.046 (3)	0.065 (4)	0.037 (3)	0.024 (3)	0.008 (3)	0.021 (3)
C11	0.043 (3)	0.050 (3)	0.037 (3)	0.011 (3)	0.014 (2)	0.020 (3)
C12	0.047 (3)	0.048 (3)	0.030 (3)	0.016 (3)	0.013 (2)	0.015 (2)
C13	0.035 (3)	0.035 (3)	0.030 (3)	0.008 (2)	0.011 (2)	0.009 (2)
C14	0.077 (5)	0.090 (6)	0.047 (4)	0.038 (5)	0.028 (4)	0.040 (4)
C15	0.045 (3)	0.041 (3)	0.040 (3)	0.016 (3)	0.018 (3)	0.014 (2)
C16	0.050 (4)	0.044 (3)	0.043 (3)	0.014 (3)	0.017 (3)	0.017 (3)
C17	0.071 (5)	0.047 (4)	0.042 (3)	0.023 (3)	0.027 (3)	0.014 (3)
C18	0.060 (5)	0.074 (5)	0.075 (5)	0.035 (4)	0.033 (4)	0.021 (4)
C19	0.045 (4)	0.086 (6)	0.086 (6)	0.031 (4)	0.021 (4)	0.023 (5)
C20	0.046 (4)	0.055 (4)	0.053 (4)	0.020 (3)	0.013 (3)	0.014 (3)
C21	0.117 (8)	0.058 (5)	0.057 (5)	0.031 (5)	0.024 (5)	0.002 (4)
C22	0.082 (5)	0.046 (4)	0.037 (3)	0.017 (3)	0.019 (3)	0.018 (3)
C23	0.083 (5)	0.036 (3)	0.045 (4)	0.008 (3)	0.017 (4)	0.006 (3)
C24	0.058 (4)	0.046 (3)	0.035 (3)	0.007 (3)	0.014 (3)	0.002 (3)
C25	0.085 (5)	0.052 (4)	0.037 (3)	0.013 (4)	0.023 (3)	0.017 (3)
C26	0.058 (4)	0.044 (3)	0.035 (3)	0.005 (3)	0.011 (3)	0.014 (3)
C27	0.098 (7)	0.063 (5)	0.040 (4)	0.014 (4)	0.027 (4)	0.004 (3)
C28	0.060 (4)	0.043 (3)	0.044 (3)	0.012 (3)	0.019 (3)	0.016 (3)
C29	0.051 (4)	0.057 (4)	0.044 (3)	0.002 (3)	0.013 (3)	0.020 (3)
C30	0.058 (4)	0.073 (5)	0.063 (5)	0.022 (4)	0.032 (4)	0.034 (4)
C31	0.099 (7)	0.058 (5)	0.080 (6)	0.018 (5)	0.025 (5)	0.039 (5)
C32	0.108 (8)	0.053 (5)	0.085 (7)	−0.006 (5)	0.011 (6)	0.031 (5)
C33	0.060 (4)	0.054 (4)	0.056 (4)	0.001 (3)	0.005 (3)	0.016 (3)
C34	0.065 (5)	0.130 (9)	0.066 (5)	0.033 (6)	0.019 (4)	0.045 (6)

### Geometric parameters (Å, °)

**Table:**

Cu1—O1	1.985 (4)	C10—H10	0.9300
Cu1—O4	1.961 (5)	C11—C12	1.384 (8)
Cu1—O6	1.963 (4)	C11—C14	1.511 (9)
Cu1—O8	1.956 (5)	C12—C13	1.398 (8)
Cu1—N1	2.215 (5)	C12—H12	0.9300
Cu1—Cu2	2.6576 (9)	C14—H14A	0.9600
Cu2—O2	1.968 (4)	C14—H14B	0.9600
Cu2—O3	2.001 (5)	C14—H14C	0.9600
Cu2—O5	1.957 (5)	C15—C20	1.369 (9)
Cu2—O7	1.981 (5)	C15—C16	1.380 (9)
Cu2—N3	2.208 (5)	C16—C17	1.391 (9)
N1—C9	1.345 (8)	C16—H16	0.9300
N1—C13	1.362 (7)	C17—C18	1.382 (11)
N2—C13	1.357 (7)	C17—C21	1.515 (10)
N2—C15	1.418 (7)	C18—C19	1.384 (12)
N2—H2	0.8600	C18—H18	0.9300
N3—C26	1.344 (8)	C19—C20	1.384 (10)
N3—C22	1.325 (9)	C19—H19	0.9300
N4—C26	1.386 (9)	C20—H20	0.9300
N4—C28	1.409 (9)	C21—H21A	0.9600
N4—H4	0.8600	C21—H21B	0.9600
O1—C1	1.259 (7)	C21—H21C	0.9600
O2—C1	1.236 (7)	C22—C23	1.376 (9)
O3—C3	1.255 (8)	C22—H22	0.9300
O4—C3	1.245 (8)	C23—C24	1.379 (10)
O5—C5	1.254 (8)	C23—H23	0.9300
O6—C5	1.262 (7)	C24—C25	1.373 (10)
O7—C7	1.248 (8)	C24—C27	1.503 (9)
O8—C7	1.251 (8)	C25—C26	1.397 (9)
C1—C2	1.501 (8)	C25—H25	0.9300
C2—H2A	0.9600	C27—H27A	0.9600
C2—H2B	0.9600	C27—H27B	0.9600
C2—H2C	0.9600	C27—H27C	0.9600
C3—C4	1.513 (9)	C28—C29	1.373 (10)
C4—H4A	0.9600	C28—C33	1.367 (10)
C4—H4B	0.9600	C29—C30	1.413 (10)
C4—H4C	0.9600	C29—H29	0.9300
C5—C6	1.503 (9)	C30—C31	1.360 (12)
C6—H6A	0.9600	C30—C34	1.464 (12)
C6—H6B	0.9600	C31—C32	1.390 (14)
C6—H6C	0.9600	C31—H31	0.9300
C7—C8	1.509 (9)	C32—C33	1.371 (12)
C8—H8A	0.9600	C32—H32	0.9300
C8—H8B	0.9600	C33—H33	0.9300
C8—H8C	0.9600	C34—H34A	0.9600
C9—C10	1.362 (9)	C34—H34B	0.9600
C9—H9	0.9300	C34—H34C	0.9600
C10—C11	1.390 (9)		
			
O8—Cu1—O4	168.87 (19)	C10—C9—H9	117.5
O8—Cu1—O6	90.3 (2)	C9—C10—C11	118.7 (6)
O4—Cu1—O6	89.0 (2)	C9—C10—H10	120.6
O8—Cu1—O1	89.5 (2)	C11—C10—H10	120.6
O4—Cu1—O1	88.3 (2)	C10—C11—C12	118.1 (6)
O6—Cu1—O1	165.03 (19)	C10—C11—C14	120.8 (6)
O8—Cu1—N1	90.72 (19)	C12—C11—C14	121.0 (6)
O4—Cu1—N1	100.40 (19)	C11—C12—C13	120.0 (5)
O6—Cu1—N1	96.21 (18)	C11—C12—H12	120.0
O1—Cu1—N1	98.76 (18)	C13—C12—H12	120.0
O8—Cu1—Cu2	84.27 (14)	N1—C13—N2	114.9 (5)
O4—Cu1—Cu2	84.61 (14)	N1—C13—C12	121.6 (5)
O6—Cu1—Cu2	83.56 (13)	N2—C13—C12	123.5 (5)
O1—Cu1—Cu2	81.53 (13)	C11—C14—H14A	109.5
N1—Cu1—Cu2	174.98 (14)	C11—C14—H14B	109.5
O5—Cu2—O2	168.97 (19)	H14A—C14—H14B	109.5
O5—Cu2—O7	89.7 (2)	C11—C14—H14C	109.5
O2—Cu2—O7	90.7 (2)	H14A—C14—H14C	109.5
O5—Cu2—O3	90.1 (2)	H14B—C14—H14C	109.5
O2—Cu2—O3	86.8 (2)	C20—C15—C16	119.8 (6)
O7—Cu2—O3	165.6 (2)	C20—C15—N2	118.6 (6)
O5—Cu2—N3	90.5 (2)	C16—C15—N2	121.5 (6)
O2—Cu2—N3	100.4 (2)	C15—C16—C17	120.7 (7)
O7—Cu2—N3	95.3 (2)	C15—C16—H16	119.6
O3—Cu2—N3	99.0 (2)	C17—C16—H16	119.6
O5—Cu2—Cu1	83.70 (13)	C18—C17—C16	118.5 (7)
O2—Cu2—Cu1	85.41 (13)	C18—C17—C21	120.6 (7)
O7—Cu2—Cu1	82.92 (14)	C16—C17—C21	120.9 (8)
O3—Cu2—Cu1	82.78 (14)	C17—C18—C19	121.2 (7)
N3—Cu2—Cu1	173.93 (14)	C17—C18—H18	119.4
C9—N1—C13	116.6 (5)	C19—C18—H18	119.4
C9—N1—Cu1	113.6 (4)	C20—C19—C18	119.0 (8)
C13—N1—Cu1	129.2 (4)	C20—C19—H19	120.5
C13—N2—C15	127.8 (5)	C18—C19—H19	120.5
C13—N2—H2	116.1	C15—C20—C19	120.8 (7)
C15—N2—H2	116.1	C15—C20—H20	119.6
C26—N3—C22	116.8 (6)	C19—C20—H20	119.6
C26—N3—Cu2	127.7 (4)	C17—C21—H21A	109.5
C22—N3—Cu2	115.2 (4)	C17—C21—H21B	109.5
C26—N4—C28	127.2 (6)	H21A—C21—H21B	109.5
C26—N4—H4	116.4	C17—C21—H21C	109.5
C28—N4—H4	116.4	H21A—C21—H21C	109.5
C1—O1—Cu1	125.2 (4)	H21B—C21—H21C	109.5
C1—O2—Cu2	122.2 (4)	N3—C22—C23	124.4 (6)
C3—O3—Cu2	123.2 (4)	N3—C22—H22	117.8
C3—O4—Cu1	123.4 (4)	C23—C22—H22	117.8
C5—O5—Cu2	124.1 (4)	C22—C23—C24	119.2 (6)
C5—O6—Cu1	123.7 (4)	C22—C23—H23	120.4
C7—O7—Cu2	123.7 (4)	C24—C23—H23	120.4
C7—O8—Cu1	123.3 (4)	C23—C24—C25	117.4 (6)
O2—C1—O1	125.1 (6)	C23—C24—C27	121.8 (7)
O2—C1—C2	118.5 (6)	C25—C24—C27	120.8 (7)
O1—C1—C2	116.4 (6)	C24—C25—C26	120.1 (7)
C1—C2—H2A	109.5	C24—C25—H25	120.0
C1—C2—H2B	109.5	C26—C25—H25	120.0
H2A—C2—H2B	109.5	N3—C26—N4	115.7 (6)
C1—C2—H2C	109.5	N3—C26—C25	122.0 (6)
H2A—C2—H2C	109.5	N4—C26—C25	122.2 (6)
H2B—C2—H2C	109.5	C24—C27—H27A	109.5
O4—C3—O3	125.7 (6)	C24—C27—H27B	109.5
O4—C3—C4	116.8 (6)	H27A—C27—H27B	109.5
O3—C3—C4	117.5 (6)	C24—C27—H27C	109.5
C3—C4—H4A	109.5	H27A—C27—H27C	109.5
C3—C4—H4B	109.5	H27B—C27—H27C	109.5
H4A—C4—H4B	109.5	C29—C28—C33	119.7 (7)
C3—C4—H4C	109.5	C29—C28—N4	121.9 (7)
H4A—C4—H4C	109.5	C33—C28—N4	118.3 (7)
H4B—C4—H4C	109.5	C28—C29—C30	121.5 (7)
O5—C5—O6	124.5 (6)	C28—C29—H29	119.3
O5—C5—C6	118.9 (6)	C30—C29—H29	119.3
O6—C5—C6	116.6 (6)	C31—C30—C29	117.4 (8)
C5—C6—H6A	109.5	C31—C30—C34	123.1 (8)
C5—C6—H6B	109.5	C29—C30—C34	119.5 (8)
H6A—C6—H6B	109.5	C30—C31—C32	121.1 (8)
C5—C6—H6C	109.5	C30—C31—H31	119.4
H6A—C6—H6C	109.5	C32—C31—H31	119.4
H6B—C6—H6C	109.5	C33—C32—C31	120.5 (8)
O7—C7—O8	125.5 (6)	C33—C32—H32	119.7
O7—C7—C8	117.3 (6)	C31—C32—H32	119.7
O8—C7—C8	117.1 (7)	C32—C33—C28	119.8 (8)
C7—C8—H8A	109.5	C32—C33—H33	120.1
C7—C8—H8B	109.5	C28—C33—H33	120.1
H8A—C8—H8B	109.5	C30—C34—H34A	109.5
C7—C8—H8C	109.5	C30—C34—H34B	109.5
H8A—C8—H8C	109.5	H34A—C34—H34B	109.5
H8B—C8—H8C	109.5	C30—C34—H34C	109.5
N1—C9—C10	124.9 (6)	H34A—C34—H34C	109.5
N1—C9—H9	117.5	H34B—C34—H34C	109.5
			
O8—Cu1—Cu2—O5	−86.6 (2)	Cu2—Cu1—O8—C7	−4.0 (5)
O4—Cu1—Cu2—O5	93.9 (2)	Cu2—O2—C1—O1	−0.5 (10)
O6—Cu1—Cu2—O5	4.4 (2)	Cu2—O2—C1—C2	−179.9 (5)
O1—Cu1—Cu2—O5	−177.0 (2)	Cu1—O1—C1—O2	7.4 (10)
O8—Cu1—Cu2—O2	95.1 (2)	Cu1—O1—C1—C2	−173.1 (5)
O4—Cu1—Cu2—O2	−84.3 (2)	Cu1—O4—C3—O3	−0.7 (9)
O6—Cu1—Cu2—O2	−173.9 (2)	Cu1—O4—C3—C4	179.5 (5)
O1—Cu1—Cu2—O2	4.8 (2)	Cu2—O3—C3—O4	5.0 (9)
O8—Cu1—Cu2—O7	3.9 (2)	Cu2—O3—C3—C4	−175.2 (5)
O4—Cu1—Cu2—O7	−175.6 (2)	Cu2—O5—C5—O6	2.5 (9)
O6—Cu1—Cu2—O7	94.8 (2)	Cu2—O5—C5—C6	−178.3 (5)
O1—Cu1—Cu2—O7	−86.5 (2)	Cu1—O6—C5—O5	3.7 (10)
O8—Cu1—Cu2—O3	−177.5 (2)	Cu1—O6—C5—C6	−175.5 (5)
O4—Cu1—Cu2—O3	3.0 (2)	Cu2—O7—C7—O8	4.3 (9)
O6—Cu1—Cu2—O3	−86.6 (2)	Cu2—O7—C7—C8	−176.4 (4)
O1—Cu1—Cu2—O3	92.1 (2)	Cu1—O8—C7—O7	1.2 (9)
O8—Cu1—N1—C9	73.9 (5)	Cu1—O8—C7—C8	−178.1 (4)
O4—Cu1—N1—C9	−106.6 (5)	C13—N1—C9—C10	−1.2 (10)
O6—Cu1—N1—C9	−16.5 (5)	Cu1—N1—C9—C10	−172.9 (6)
O1—Cu1—N1—C9	163.6 (5)	N1—C9—C10—C11	1.0 (12)
O8—Cu1—N1—C13	−96.5 (5)	C9—C10—C11—C12	−0.7 (10)
O4—Cu1—N1—C13	83.0 (5)	C9—C10—C11—C14	177.8 (7)
O6—Cu1—N1—C13	173.1 (5)	C10—C11—C12—C13	0.9 (10)
O1—Cu1—N1—C13	−6.9 (5)	C14—C11—C12—C13	−177.6 (7)
O5—Cu2—N3—C26	−117.0 (6)	C9—N1—C13—N2	−175.8 (6)
O2—Cu2—N3—C26	61.6 (6)	Cu1—N1—C13—N2	−5.6 (8)
O7—Cu2—N3—C26	153.3 (6)	C9—N1—C13—C12	1.3 (9)
O3—Cu2—N3—C26	−26.8 (6)	Cu1—N1—C13—C12	171.5 (4)
O5—Cu2—N3—C22	56.7 (6)	C15—N2—C13—N1	−176.0 (6)
O2—Cu2—N3—C22	−124.7 (6)	C15—N2—C13—C12	6.9 (11)
O7—Cu2—N3—C22	−33.1 (6)	C11—C12—C13—N1	−1.2 (9)
O3—Cu2—N3—C22	146.9 (5)	C11—C12—C13—N2	175.7 (6)
O8—Cu1—O1—C1	−92.0 (6)	C13—N2—C15—C20	−134.6 (7)
O4—Cu1—O1—C1	77.1 (6)	C13—N2—C15—C16	49.4 (10)
O6—Cu1—O1—C1	−2.5 (12)	C20—C15—C16—C17	−0.3 (10)
N1—Cu1—O1—C1	177.4 (5)	N2—C15—C16—C17	175.7 (6)
Cu2—Cu1—O1—C1	−7.7 (5)	C15—C16—C17—C18	−0.6 (10)
O5—Cu2—O2—C1	−13.1 (15)	C15—C16—C17—C21	−178.9 (7)
O7—Cu2—O2—C1	78.8 (5)	C16—C17—C18—C19	0.4 (13)
O3—Cu2—O2—C1	−87.0 (5)	C21—C17—C18—C19	178.7 (9)
N3—Cu2—O2—C1	174.4 (5)	C17—C18—C19—C20	0.8 (14)
Cu1—Cu2—O2—C1	−4.0 (5)	C16—C15—C20—C19	1.5 (11)
O5—Cu2—O3—C3	−88.6 (5)	N2—C15—C20—C19	−174.6 (7)
O2—Cu2—O3—C3	80.8 (5)	C18—C19—C20—C15	−1.7 (13)
O7—Cu2—O3—C3	0.7 (12)	C26—N3—C22—C23	0.6 (12)
N3—Cu2—O3—C3	−179.1 (5)	Cu2—N3—C22—C23	−173.8 (7)
Cu1—Cu2—O3—C3	−5.0 (5)	N3—C22—C23—C24	1.6 (13)
O8—Cu1—O4—C3	−5.3 (15)	C22—C23—C24—C25	−2.7 (12)
O6—Cu1—O4—C3	81.3 (5)	C22—C23—C24—C27	177.3 (8)
O1—Cu1—O4—C3	−84.0 (5)	C23—C24—C25—C26	1.8 (12)
N1—Cu1—O4—C3	177.4 (5)	C27—C24—C25—C26	−178.2 (8)
Cu2—Cu1—O4—C3	−2.4 (5)	C22—N3—C26—N4	−179.6 (7)
O2—Cu2—O5—C5	4.1 (15)	Cu2—N3—C26—N4	−6.0 (10)
O7—Cu2—O5—C5	−87.9 (5)	C22—N3—C26—C25	−1.6 (11)
O3—Cu2—O5—C5	77.7 (5)	Cu2—N3—C26—C25	172.0 (6)
N3—Cu2—O5—C5	176.8 (5)	C28—N4—C26—N3	−171.1 (7)
Cu1—Cu2—O5—C5	−5.0 (5)	C28—N4—C26—C25	10.9 (13)
O8—Cu1—O6—C5	78.6 (5)	C24—C25—C26—N3	0.4 (12)
O4—Cu1—O6—C5	−90.3 (5)	C24—C25—C26—N4	178.3 (8)
O1—Cu1—O6—C5	−10.7 (12)	C26—N4—C28—C29	44.1 (12)
N1—Cu1—O6—C5	169.4 (5)	C26—N4—C28—C33	−139.0 (8)
Cu2—Cu1—O6—C5	−5.6 (5)	C33—C28—C29—C30	−0.4 (11)
O5—Cu2—O7—C7	78.3 (5)	N4—C28—C29—C30	176.4 (7)
O2—Cu2—O7—C7	−90.7 (5)	C28—C29—C30—C31	0.5 (12)
O3—Cu2—O7—C7	−11.1 (12)	C28—C29—C30—C34	−178.3 (8)
N3—Cu2—O7—C7	168.8 (5)	C29—C30—C31—C32	−0.4 (14)
Cu1—Cu2—O7—C7	−5.4 (5)	C34—C30—C31—C32	178.4 (10)
O4—Cu1—O8—C7	−1.1 (15)	C30—C31—C32—C33	0.1 (17)
O6—Cu1—O8—C7	−87.5 (5)	C31—C32—C33—C28	0.1 (16)
O1—Cu1—O8—C7	77.5 (5)	C29—C28—C33—C32	0.0 (13)
N1—Cu1—O8—C7	176.3 (5)	N4—C28—C33—C32	−176.9 (9)

### Hydrogen-bond geometry (Å, °)

**Table:**

*D*—H···*A*	*D*—H	H···*A*	*D*···*A*	*D*—H···*A*
N2—H2···O1	0.86	1.99	2.830 (6)	165
N4—H4···O3	0.86	2.18	2.964 (8)	152
